# Expression and regulation of Angiopoietins and their receptor Tie-2 in sika deer antler

**DOI:** 10.1080/19768354.2017.1317023

**Published:** 2017-05-03

**Authors:** Hong-Liang Zhang, Zhan-Peng Yue, Lu Zhang, Zhan-Qing Yang, Shuang Geng, Kai Wang, Hai-Fan Yu, Bin Guo

**Affiliations:** College of Veterinary Medicine, Jilin University, Changchun, People’s Republic of China

**Keywords:** Angiopoietin, testosterone, estrogen, retinoic acid, antler chondrocyte

## Abstract

The cartilage vascularization and chondrocyte survival are essential for endochondral ossification which occurs in the process of antler growth. Angiopoietins (Ang) is a family of major angiogenic growth factors and involved in regulating the vascularization. However, the expression and regulation of Angs in the antler are still unknown. The aim of this study is to localize the expression of Ang-1, Ang-2 and their receptor Tie-2 in sika deer antler using *in situ* hybridization and focused on analyzing the regulation of testosterone, estrogen, all-trans-retinoic acid (ATRA) and 9cRA on their expression in antler chondrocytes. The results showed that Ang-1, Ang-2 and Tie-2 were highly expressed in antler chondrocytes. Administration of testosterone to antler chondrocytes led to a notable increase in the expression of Ang-1 and Tie-2, and a reduction in the expression of Ang-2. The similar result was also observed after estrogen treatment. In contrast, ATRA and 9cRA could inhibit the expression of Ang-1 in antler chondrocytes and heighten the expression of Ang-2. Simultaneously, ATRA could downregulate the expression of Tie-2 in antler chondrocytes at 12 and 24 h, while 9cRA upregulate the expression of Tie-2 at 3 and 6 h. Collectively, Ang-1, Ang-2 and Tie-2 are expressed in antler chondrocytes and their expression can be affected by testosterone, estrogen, ATRA and 9cRA.

## Introduction

Deer antlers are bony appendages that develop from permanent outgrowth of the frontal bone known as pedicle (Kierdorf et al. 2009). It is not only one of the most important medicines, but also a unique mammalian organ that can fully grow back once lost from its pedicle (Sui et al. 2014; Deb-Choudhury et al. 2015). Therefore, antlers probably offer the most pertinent model for studying organ regeneration in mammals. Although accumulating evidence has demonstrated that testosterone, estrogen and retinoic acid might participate in the process of antler growth, their underlying mechanisms remain poorly understood (Lincoln & Tyler 1999; Kierdorf & Bartos 1999; Li et al. 2009; Bartos et al. 2009). Antler regeneration involves endochondral ossification which includes cartilage development, calcification, removal and replacement by bone (Gomez et al. 2013; Jing et al. 2015). Moreover, the new blood vessels invasion into cartilage may be important for endochondral ossification, especially in the cartilage development (Bluteau et al. 2007). The antler cartilage zone contains columns of chondrocytes and large blood vessels which feed and nourish the rapidly growing antler (Clark et al. 2006a). Therefore, cartilage vascularization may be essential for antler regeneration. It has been previously reported that vascular endothelial growth factor (VEGF), which was a major angiogenic factor and might modulate new blood vessels invasion into the cartilage, was highly expressed in antler chondrocytes (Tink & Barnstable 2004; Clark et al. 2006b). Further evidence determined that VEGF effects were complemented and coordinated by angiopoietins (Ang) (Valable et al. 2005; Makinde et al. 2006).

Ang is involved in the maturation, stabilization and remodeling of vessels (Toyama et al. 2009). Four members of the Ang family have been discovered and the best studied are angiopoietin-1 (Ang-1) and Ang-2 which regulate the angiogenesis in the process of endochondral ossification and participate in cartilage vascularization and chondrocyte maturation (Jing et al. 2013; Kim et al. 2013). Ang-1 and Ang-2 exert their biological effects through activating or blocking the activation of their receptor tyrosine kinase with immunoglobulin and epidermal growth factor homology domains (Tie-2). Ang-1 can induce the autophosphorylation of Tie-2, promote endothelial cells survival and induce their migration and sprouting. In contrast, Ang-2 acts as a natural antagonist for Ang-1 to disrupt the angiogenesis (Maisonpierre et al. 1997). It binds to Tie-2 with a similar affinity of Ang-1, but does not induce receptor phosphorylation. Indeed, Ang-2 competitively inhibits the activation of Tie-2 receptor and plays an opposing effect with Ang-1 (Maisonpierre et al. 1997). Although previous study had showed that Ang-1 and Ang-2 were highly expressed in rabbit condylar chondrocytes (Jing et al. 2013), there were no reports whether they were also expressed in antler cartilage. The present study was undertaken to examine the expression of Ang-1, Ang-2 and Tie-2 in sika deer antler and then focused our attention on analyzing the regulation of testosterone, estrogen, all-trans-retinoic acid (ATRA) and 9-cis-retinoic acid (9cRA) on their expression in antler chondrocytes.

## Materials and methods

### Tissue collection

Antler tissues from three-year-old health sika deer as growing for about 60 days were collected as previously described (Zhang et al. 2017). Briefly, the distal 5 cm of growing tip was removed and sectioned sagittally along the longitudinal axis. A part of the tip was flash frozen in liquid nitrogen and then stored at −70°C for *in situ* hybridization. The remaining tip was used for isolation of antler chondrocytes.

### Isolation and treatment of antler chondrocytes

The antler chondrocytes from sika deer were isolated and cultured as previously described (Zhang et al. 2017). Cultured chondrocytes were treated with testosterone (10^−10^ g/ml), estradiol-17β (10^−11^ g/ml), ATRA (1 nM) or 9cRA (1 nM) for 3, 6, 12 and 24 h. Testosterone and estradiol-17β were dissolved in dimethyl sulfoxide, while ATRA and 9cRA were melted in ethanol. Controls received the vehicle only.

### *In situ* hybridization

Total RNA from antler cartilage was reverse-transcribed and amplified with the corresponding primers (Table 1). The cloned fragment of different gene was inserted into pGEM-T plasmid and then amplified with the primers for T7 and SP6 to prepare templates which were used to label antisense and sense cRNA probes according to the instructions of DIG RNA labeling kit (Roche Diagnostics GmbH, Mannheim, Germany).

**Table 1.** Primers used in this study.

Gene	Primer sequence	Size (bp)	Application
Ang-1	AACCGGATTTCTCTTCCCAG CTGCTCTGCAGTCTGAGAGAGG	200	*In situ* hybridization
Ang-2	TAAGCAGCATCAGCCAACCAG CCTGAGCCTTTCCAGTAGTACC	205	*In situ* hybridization
Tie-2	GACATTCTTCCTCCTCAACCAG AGGCCCTTGAGCTGATACTG	200	*In situ* hybridization
Ang-1	AGTCCAGAAAACGGTGGGAG TCTGCAGAGCGTTTGTGTTG	133	Real-time PCR
Ang-2	AGGGACAGCCGGCAAAATAA CCTGTGAGCATCTGTGAGCA	108	Real-time PCR
Tie-2	GGCTTGGCGGCATATTCAAG GGAGGAAGACCGATGGAAGC	165	Real-time PCR
Gapdh	GAAGGGTGGCGCCAAGAGGG GGGGGCCAAGCAGTTGGTGG	142	Real-time PCR

Frozen sections (10 μm) were mounted on 3-aminopropyltriethoxy-silane (Sigma)-coated slides and fixed in 4% paraformaldehyde solution in phosphate-buffered saline. Hybridization was performed as described previously (Li et al. 2016). The digoxigenin-labeled sense probe was used as a negative control. Endogenous alkaline phosphatase activity was inhibited with 2 mM levamisole. All of the sections were counterstained with 1% methyl green. The positive signal was visualized as a dark brown color.

### Real-time PCR

Total RNAs from cultured cells were isolated using TRIPURE reagent (Roche) according to the manufacturer’s protocol and reverse-transcribed into cDNA using moloney murine leukemia virus reverse-transcriptase (Promega). The expression levels of different genes were validated by real-time PCR analysis using FS Universal SYBR Green Real Master (Roche) on BIO-RAD CFX96^TM^ Real-Time Detection System according to manufacturer’s instructions. Data were normalized to Gapdh expression and analyzed using the 2^−ΔΔCt^ method. All reactions were repeated at least three times. Primer sequences used for real-time PCR were listed in Table 1.

### Statistics

All data were presented as mean ± SEM. Each experiment was independently repeated at least three times. The significance of difference was analyzed by Independent-Samples *T*-Test. A *P*-value <0.05 was considered as statistically significant (*P < 0.05).

## Results

### Expression of Ang-1, Ang-2 and Tie-2 mRNA in sika deer antler

**Figure 1.**
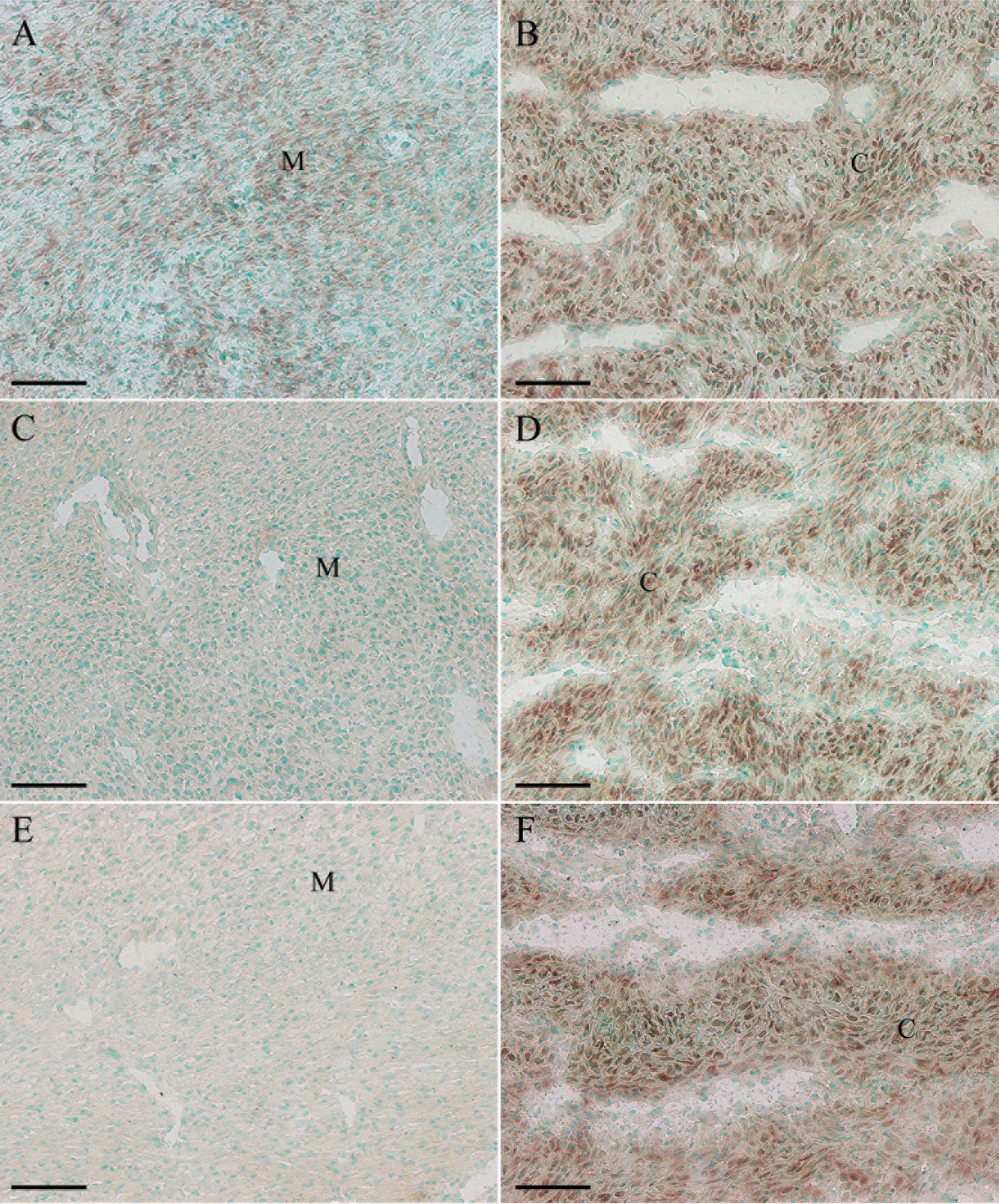
*In situ* hybridization of Ang-1, Ang-2 and Tie-2 expression in sika deer antler. (A), Ang-1 expression in antler mesenchyme. (B), Ang-1 expression in antler cartilage. (C), Ang-2 expression in antler mesenchyme. (D), Ang-2 expression in antler cartilage. (E), Tie-2 expression in antler mesenchyme. (F), Tie-2 expression in antler cartilage. M, mesenchymal cells; C, chondrocytes. Bar = 60 μm.

*In situ* hybridization was used to localize the distribution of Ang-1, Ang-2 and Tie-2 mRNA in sika deer antler. The results showed that Ang-1 mRNA was localized in mesenchymal cells (Figure 1(A)). Simultaneously, a significantly high level of Ang-1 expression was noted in antler chondrocytes (Figure 1(B)). Ang-2 expression was visualized in the chondrocytes, while no visible signal was found in mesenchymal cells (Figure 1(C,D)). The expression pattern of Tie-2 mRNA in the antler was very similar to that of Ang-2 (Figure 1(E,F)).

### Effects of testosterone on the expression of Ang-1, Ang-2 and Tie-2 mRNA in antler chondrocytes

**Figure 2.**
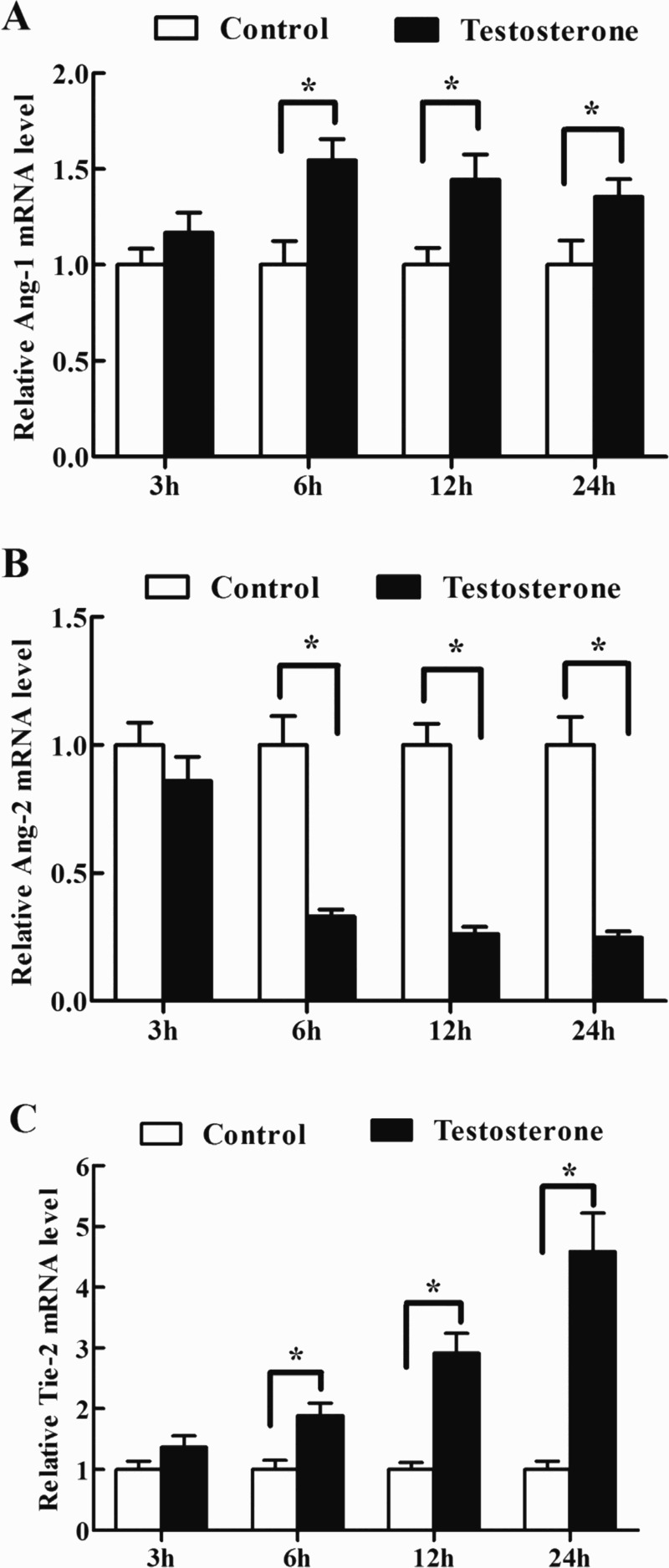
Effects of testosterone on the expression of Ang-1, Ang-2 and Tie-2 in antler chondrocytes. After antler chondrocytes were treated with testosterone for 3, 6, 12 and 24 h, the expression of Ang-1 (A), Ang-2 (B) and Tie-2 (C) was determined by real-time PCR. Data are shown mean ± SEM. Asterisks denote significance (*P* < 0.05).

In antler chondrocytes, testosterone could significantly induce the expression of Ang-1 mRNA at 6, 12 and 24 h (Table 2, Figure 2(A)). In contrast, administration of testosterone to the chondrocytes led to a notable reduction in the expression of Ang-2, which reached a nadir at 24 h (Table 2, Figure 2(B)). In the meantime, a gradual increase in the level of Tie-2 mRNA was observed between 3 and 24 h and reached the highest level at 24 h (Table 2, Figure 2(C)).

**Table 2.** Effects of testosterone on the expression of Ang-1, Ang-2 and Tie-2 mRNA in antler chondrocytes.

	3 h		6 h		12 h		24 h	
Gene	Control	Testosterone	Control	Testosterone	Control	Testosterone	Control	Testosterone
Ang-1	1.000 ± 0.085	1.173 ± 0.103	1.000 ± 0.123	1.546 ± 0.110*	1.000 ± 0.089	1.445 ± 0.131*	1.000 ± 0.128	1.354 ± 0.095*
Ang-2	1.000 ± 0.087	1.055 ± 0.104	1.000 ± 0.113	0.331 ± 0.028*	1.000 ± 0.084	0.262 ± 0.029*	1.000 ± 0.110	0.248 ± 0.024*
Tie-2	1.000 ± 0.140	1.367 ± 0.187	1.000 ± 0.151	1.888 ± 0.204*	1.000 ± 0.114	2.914 ± 0.328*	1.000 ± 0.140	4.587 ± 0.634*

### Effects of estrogen on Ang-1, Ang-2 and Tie-2 mRNA expression in antler chondrocytes

**Figure 3.**
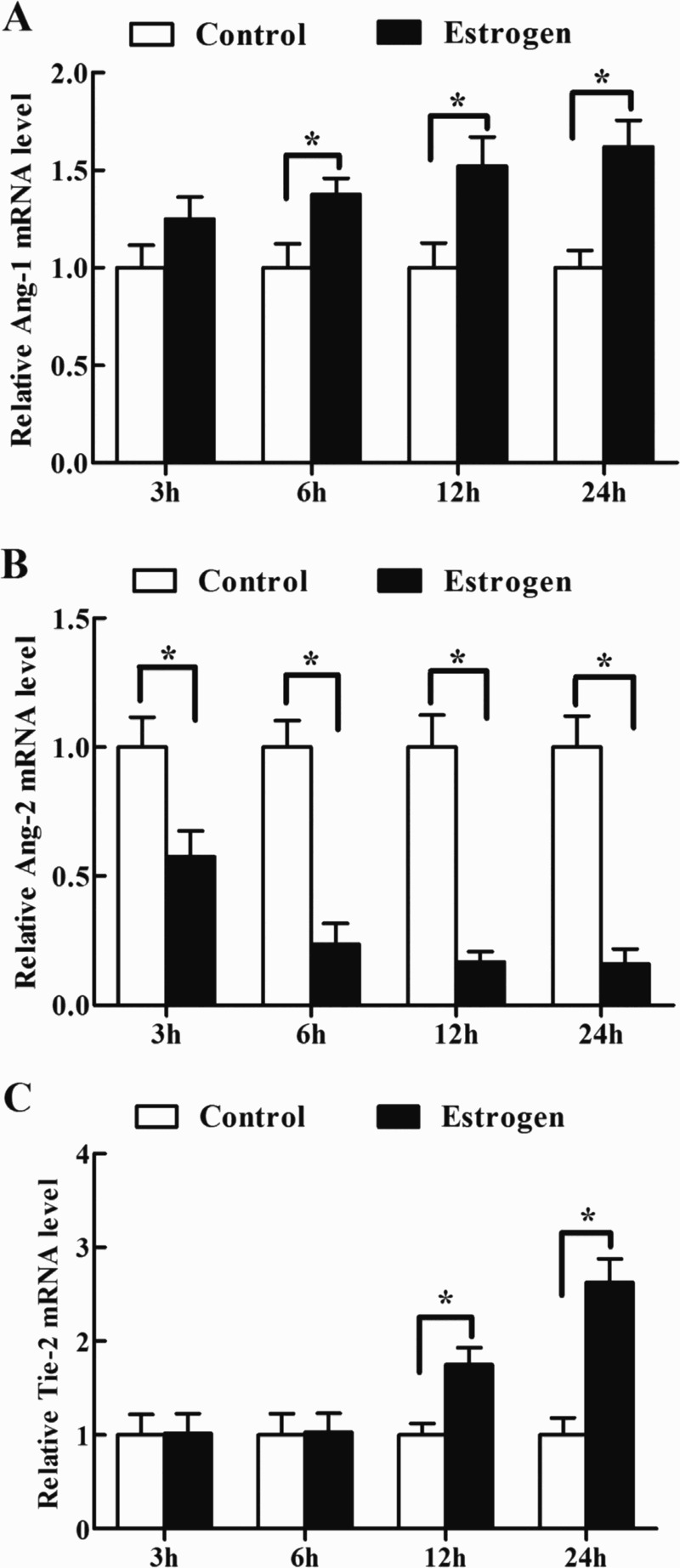
Effects of estrogen on the expression of Ang-1, Ang-2 and Tie-2 in antler chondrocytes. After antler chondrocytes were treated with estrogen for 3, 6, 12 and 24 h, the expression of Ang-1 (A), Ang-2 (B) and Tie-2 (C) was determined by real-time PCR.

To evaluate the regulation of estrogen on the expression of Ang-1, Ang-2 and Tie-2, antler chondrocytes were treated with estrogen for 3, 6, 12 and 24 h. The results found that a gradual increase in the level of Ang-1 mRNA was observed between 3 and 24 h after estrogen treatment and reached a peak at 24 h (Table 3, Figure 3(A)). Conversely, Ang-2 mRNA expression was gradually decreased and reached a nadir at 24 h (Table 3, Figure 3(B)). After antler chondrocytes were treated with estrogen, Tie-2 mRNA expression was obviously enhanced at 12 and 24 h (Table 3, Figure 3(C)).

**Table 3.** Effects of estrogen on the expression of Ang-1, Ang-2 and Tie-2 mRNA in antler chondrocytes.

	3 h		6 h		12 h		24 h	
Gene	Control	Estrogen	Control	Estrogen	Control	Estrogen	Control	Estrogen
Ang-1	1.000 ± 0.116	1.254 ± 0.112	1.000 ± 0.123	1.376 ± 0.083*	1.000 ± 0.128	1.521 ± 0.149*	1.000 ± 0.089	1.621 ± 0.135*
Ang-2	1.000 ± 0.117	0.574 ± 0.102*	1.000 ± 0.103	0.236 ± 0.081*	1.000 ± 0.124	0.167 ± 0.041*	1.000 ± 0.121	0.179 ± 0.059*
Tie-2	1.000 ± 0.219	1.020 ± 0.207	1.000 ± 0.228	1.029 ± 0.202	1.000 ± 0.123	1.751 ± 0.180*	1.000 ± 0.181	2.629 ± 0.250*

### Effects of ATRA on Ang-1, Ang-2 and Tie-2 mRNA expression in antler chondrocytes

**Figure 4.**
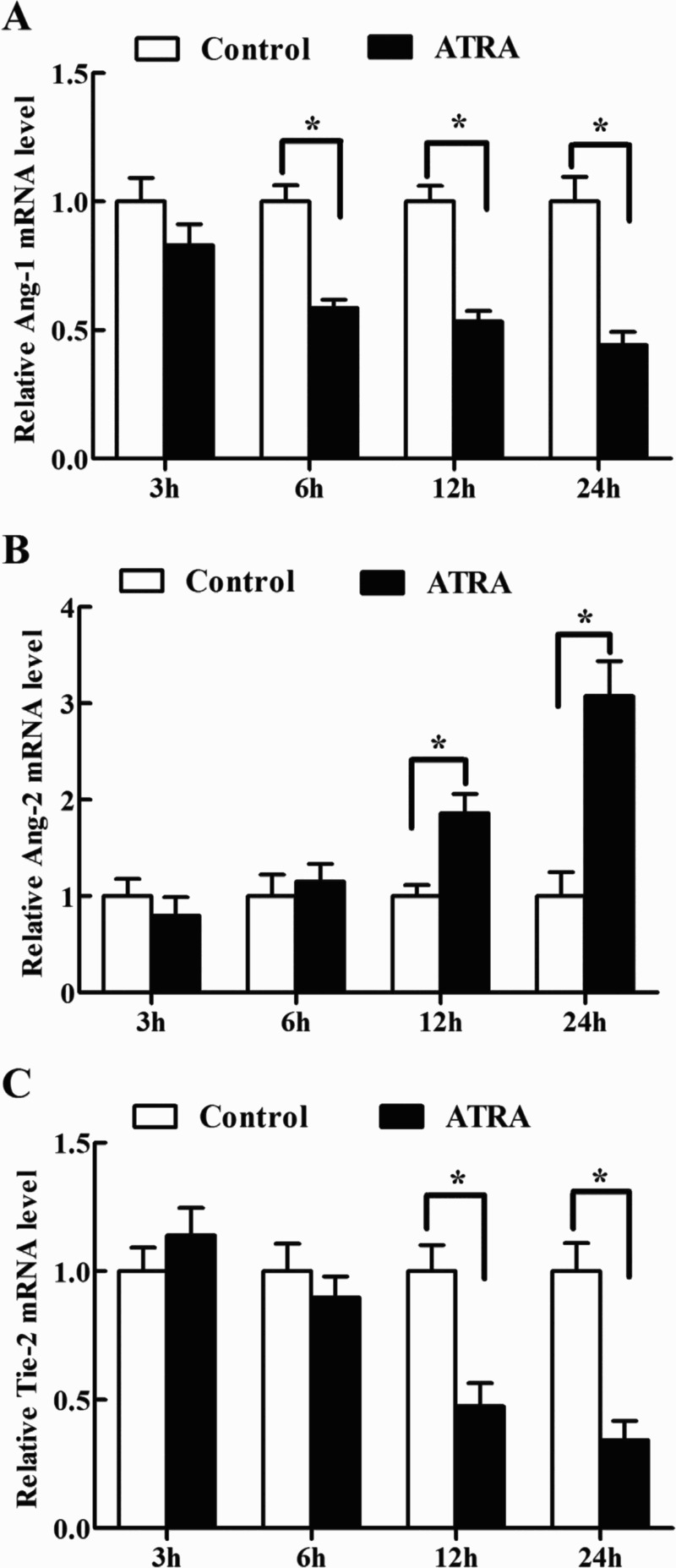
Effects of ATRA on the expression of Ang-1, Ang-2 and Tie-2 in antler chondrocytes. After antler chondrocytes were treated with ATRA for 3, 6, 12 and 24 h, the expression of Ang-1 (A), Ang-2 (B) and Tie-2 (C) was determined by real-time PCR.

Because ATRA is considered to be the biologically active isomer of RA, we treated antler chondrocytes with ATRA to see its regulation on the expression of Ang-1, Ang-2 and Tie-2. The result revealed that ATRA could time-dependently inhibit the expression of Ang-1 which reached the minimum at 24 h, and heighten the expression of Ang-2 at 12 and 24 h (Table 4, Figure 4(A,B)). In the meantime, ATRA treatment resulted in a marked decline in the expression of Tie-2 at 12 and 24 h (Table 4, Figure 4(C)).

**Table 4.** Effects of ATRA on the expression of Ang-1, Ang-2 and Tie-2 mRNA in antler chondrocytes.

	3 h		6 h		12 h		24 h	
Gene	Control	ATRA	Control	ATRA	Control	ATRA	Control	ATRA
Ang-1	1.000 ± 0.115	0.831 ± 0.106	1.000 ± 0.063	0.586 ± 0.032*	1.000 ± 0.061	0.533 ± 0.041*	1.000 ± 0.097	0.442 ± 0.051*
Ang-2	1.000 ± 0.177	0.799 ± 0.188	1.000 ± 0.222	1.153 ± 0.183	1.000 ± 0.116	1.860 ± 0.202*	1.000 ± 0.249	3.076 ± 0.363*
Tie-2	1.000 ± 0.121	1.140 ± 0.107	1.000 ± 0.127	0.808 ± 0.117	1.000 ± 0.120	0.475 ± 0.108*	1.000 ± 0.119	0.343 ± 0.105*

### Effects of 9cRA on Ang-1, Ang-2 and Tie-2 mRNA expression in antler chondrocytes

**Figure 5.**
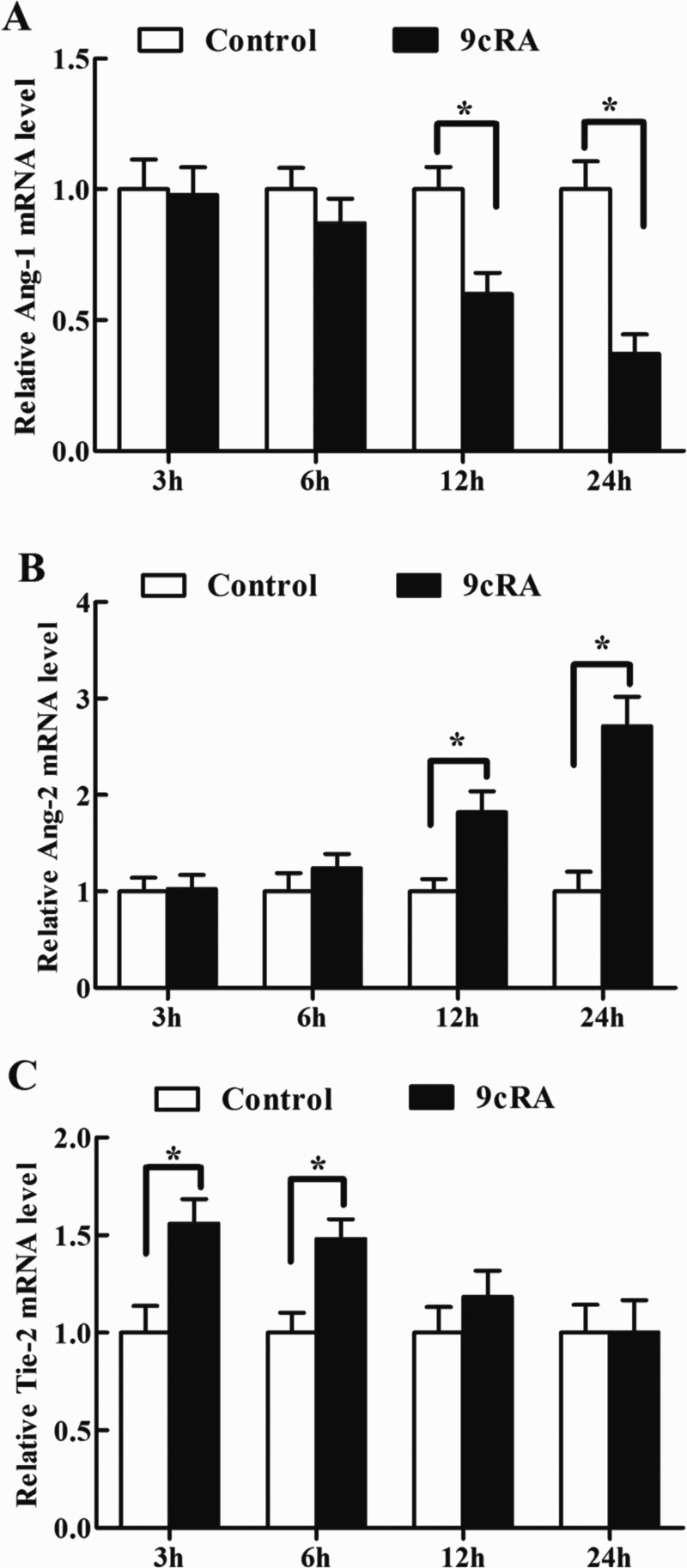
Effects of 9cRA on the expression of Ang-1, Ang-2 and Tie-2 in antler chondrocytes. After antler chondrocytes were treated with 9cRA for 3, 6, 12 and 24 h, the expression of Ang-1 (A), Ang-2 (B) and Tie-2 (C) was determined by real-time PCR.

Administration of 9cRA to antler chondrocytes caused downregulation of Ang-1 expression at 12 and 24 h (Table 5, Figure 5(A)). On the contrary, the levels of Ang-2 mRNA showed an increase at 12 and 24 h after 9cRA treatment (Table 5, Figure 5(B)). Interestingly, Tie-2 expression was gradually decreased and reached a level similar to that of the control at 24 h, although 9cRA could stimulate the expression of Tie-2 in antler chondrocytes at 3 and 6 h compared with that in the control cells (Table 5, Figure 5(C)).

**Table 5.** Effects of 9cRA on the expression of Ang-1, Ang-2 and Tie-2 mRNA in antler chondrocytes.

	3 h		6 h		12 h		24 h	
Gene	Control	9cRA	Control	9cRA	Control	9cRA	Control	9cRA
Ang-1	1.000 ± 0.114	0.979 ± 0.105	1.000 ± 0.082	0.871 ± 0.094	1.000 ± 0.086	0.599 ± 0.082*	1.000 ± 0.107	0.371 ± 0.075*
Ang-2	1.000 ± 0.243	1.029 ± 0.269	1.000 ± 0.291	1.240 ± 0.250	1.000 ± 0.227	1.821 ± 0.219*	1.000 ± 0.204	2.711 ± 0.306*
Tie-2	1.000 ± 0.137	1.560 ± 0.125*	1.000 ± 0.103	1.476 ± 0.101*	1.000 ± 0.133	1.185 ± 0.132	1.000 ± 0.143	1.003 ± 0.164

## Discussion

The Ang family has been confirmed to be involved in vasculature development and angiogenesis (Daponte et al. 2013). In this study, we examined the expression of Ang-1, Ang-2 and Tie-2 in sika deer antler using *in situ* hybridization. The results revealed that Ang-1 mRNA was highly expressed in antler chondrocytes. This was consistent with the previous evidence that VEGF mRNA signal was observed in antler chondrocytes (Clark et al. 2006b). Based on the expression pattern of VEGF and Ang-1 in antler cartilage, we conjectured that they might play a similar role of promoting new blood vessel invasion into cartilage and keeping chondrocytes survival. Meanwhile, Ang-1 might activate Tie-2 which was also highly expressed in antler chondrocytes, and further stimulate blood vessel invasion into hypertrophic zone of cartilage (Jing et al. 2013; Kim et al. 2013). On the contrary, Ang-2 was a naturally occurring antagonist of Ang-1 and could competitively inhibit Ang-1-induced activation of Tie-2 (Escobar et al. 2004). The present study found that Ang-2 was visualized in antler chondrocytes, implying that Ang-2 might antagonize the role of Ang-1, prevent blood vessels excessive invasion and induce chondrocyte apoptosis. The viewpoint was consistent with the evidence that Ang-2 could exert the foundation of inducing endothelial cells and astrocyte apoptosis and vessel regression (Lobov et al. 2002; Oshima et al. 2005; Yun et al. 2016).

It is well established that testosterone is crucial for antler growth. Castration of prepubertal male deer abolishes further pedicle and antler formation, while castration of adult males prevents full antler calcification. Moreover, administration of exogenous testosterone could overcome the abnormalities of castration (Li et al. 2009). In antler chondrocytes, testosterone could promote the expression of Ang-1 and Tie-2, and inhibit the expression of Ang-2, suggesting that testosterone might regulate cartilage vascularization and chondrocytes survival in growing antler through Ang-1, Ang-2 and Tie-2. This evidence was consistent with the previous finding that dihydrotestosterone could enhance neovascularization in the context of ischemic injury of the male mouse and induce endothelial cell survival (Sieveking et al. 2010; Cai et al. 2011).

It has previously showed that testosterone could convert into estrogen which was the most potent steroid causing maturation and mineralization of antlers in intact and castrated deer (Lincoln & Tyler 1999; Bubenik et al. 2005). In buck antler, estrogen could also be compounded (Kierdorf et al. 2009). Moreover, estrogen could upregulate the expression of Ang-1 and Tie-2 in antler chondrocytes, and downregulate the expression of Ang-2, indicating that estrogen might produce the similar biological effects with that of testosterone. Indeed, estrogen could induce angiogenesis in adenomyosis development via activating the E2-Slug-VEGF pathway (Huang et al. 2014).

RA is the biologically active metabolite of vitamin A and exists as several geometric isomers including ATRA, 9cRA, 13cRA, etc. (Thatcher & Isoherranen 2009). Injection of ATRA into the growing pedicle led to a significant increase in first antler volume (Kierdorf & Bartos 1999), suggesting a role of ATRA in antler growth. The present study showed that ATRA could induce the expression of Ang-2, and suppress the expression of Ang-1 and Tie-2, demonstrating that ATRA might play a role in preventing cartilage excessive vascularization in the antler growth. The notion was further confirmed in cancer cells (Kini et al. 2001; Hoffmann et al. 2007; Vourtsis et al. 2013; Liang et al. 2014). 9cRA also appears to have biological activity and has been used for treatment of Kaposi’s sarcoma and chronic hand eczema (Nelson et al. 2013). In antler chondrocytes, regulation of Ang-1, Ang-2 and Tie-2 by 9cRA reveals a role of 9cRA in cartilage vascularization. Together these data evidenced that ATRA and 9cRA might synergistically inhibit the vascularization of antler cartilage and seem to have an antagonistic role of testosterone and estrogen. Indeed, in human microvascular endothelial cells, ATRA and 9cRA might withstand the effect of testosterone on the formation of capillary-like tubular structures (Lansink et al., 1998). Meanwhile, the inhibitory action of RA on the survival of breast cancer cell also could be antagonized by estrogen (Saumet et al., 2012). These results suggested that ATRA and 9cRA might govern the vascularization of antler cartilage together with estrogen and testosterone to maintain normal antler growth.

In summary, Ang-1, Ang-2 and Tie-2 are expressed in antler chondrocytes and their expression can be affected by testosterone, estrogen, ATRA and 9cRA.
